# Supplementary material for: Hollow mesoporous atomically dispersed metal-nitrogen-carbon catalysts with enhanced diffusion for catalysis involving larger molecules
Source: Nat Commun. 2022 May 24;13:2900. doi: 10.1038/s41467-022-30520-3 (PMC9130124; doi:10.1038/s41467-022-30520-3)
Supplement: Supplementary file 1 — Supplementary Information [file 41467_2022_30520_MOESM1_ESM.pdf]

Supplementary Information

Hollow mesoporous atomically dispersed metal-nitrogen-carbon catalysts with enhanced diffusion for catalysis involving larger molecules

Xu Han[1+], *Tianyu Zhang*[1+], *Xinhe Wang*[1], *Zedong Zhang*[1], *Yaping Li*[1], *Yongji Qin*[1], *Bingqing Wang*[1], *Aijuan Han*[1*], *Junfeng Liu*[1*]

[1]State Key Laboratory of Chemical Resource Engineering, Beijing University of Chemical Technology, Beijing 100029, China

E-mail: ljf@mail.buct.edu.cn; hanaijuan@mail.buct.edu.cn

[+] These authors contributed equally to this work.

Contents

Supplementary Notes 3
Supplementary Figures 6
Supplementary Tables 14
Supplementary References 17

Supplementary Notes

Chemicals: Cobalt (II) acetate tetrahydrate ($Co(CH_3COO)_2 \cdot 4H_2O$), zinc acetate dihydrate ($Zn(CH_3COO)_2 \cdot 2H_2O$), nickel (II) acetate tetrahydrate ($Ni(CH_3COO)_2 \cdot 4H_2O$), manganese (II) acetate tetrahydrate ($Mn(CH_3COO)_2 \cdot 4H_2O$), copper (II) acetate monohydrate ($Cu(CH_3COO)_2 \cdot H_2O$) were purchased from Beijing Xilong Chemical Factory. 2-methylimidazole (2-MIM) was purchased from Shandong Xiya Chemical Factory Co., Ltd. Cetyl-trimethyl ammonium bromide (CTAB) was purchased from Tianjin Jinke Fine Chemical Research Institute. Tannic acid (TA, purity>96%) was purchased from Tianjin Heowns Biochemical Technology Co., Ltd. Aniline, 4-chloroaniline and 4-methoxyaniline was purchased from Aladdin Reagent Co., LTD. Dimethyl sulfoxide (DMSO), potassium hydroxide (KOH, 83 wt.%) and methanol (CH_3OH) were purchased from Beijing Chemical Works. All chemical reagents were A.R. grade and used as received without further purification.

Synthesis of h-Co_{NP}NC: The synthesis of h-Co_{NP}NC was similar to that of h-CoNC, except $Zn(CH_3COO)_2 \cdot 2H_2O$ (285.7 mg, 1.30 mmol) and $Co(CH_3COO)_2 \cdot 4H_2O$ (16.2 mg, 0.065 mmol) with a molar ratio of 20:1 were used as precursors.

Synthesis of s-CoNC: The synthesis of s-CoNC was similar to that of h-CoNC, except solid ZnCo-BZIF without etching process was used as a precursor to anneal in the tube furnace.

Synthesis of h-NC: The synthesis of h-NC was similar to that of h-CoNC, except only 300.0 mg of $Zn(CH_3COO)_2 \cdot 2H_2O$ was used as the reactant.

Characterizations: The morphologies and sizes of samples were characterized by a transmission electron microscope (TEM, JEM-2100F) operated at an acceleration voltage of 200 kV and a field emission scanning electron microscope (SEM, Nova NanoSEM 450, FEI). Aberration corrected high-angle annular dark-field scanning transmission electron microscopy (AC-HAADF-STEM) was carried out on JEM-ARM200F (JEOL, Tokyo, Japan) TEM/STEM operated at 200 kV with cold filed-emission gun and aberration corrector. X-ray photoelectron spectroscopy (XPS) measurements were conducted on a Thermo ESCALAB spectrometer using a monochromated Al Kα radiation (hv=1486.6 eV). The energy calibration of the spectrometer was performed using the C 1*s* peak at 284.8 eV. Powder X-ray diffraction

patterns (XRD) were recorded on a Bruker D8 Advance X-ray diffractometer equipped with a Cu-Kα radiation source (λ = 1.5418 Å) and operated at a scan rate of 10° min^{-1}. The inductively coupled plasma optical emission spectrometer (ICP-OES) measurements were conducted on an iCAP7600 spectrometer for metal elemental analysis.

XAFS measurement: AFS spectra at the Co K-edge, Ni K-edge, Mn K-edge, and Cu K-edge were measured at BL14W1 station in Shanghai Synchrotron Radiation Facility (SSRF) and the BL1W1B station at Beijing Synchrotron Radiation Facility (BSRF). The Co K-edge XANES data of h-CoNC, Ni K-edge XANES data of h-NiNC, Mn K-edge XANES data of h-MnNC, and Cu K-edge XANES data of h-CuNC were recorded in a fluorescence mode, while the corresponding references were recorded in a transmission mode. The storage ring was operated at the energy of 2.5 GeV with an average electron current of 250 mA. The hard X-ray was monochromatized with Si (111) double-crystals. The obtained extended X-ray absorption fine structure (EXAFS) data were processed with the ATHENA module. The k^3-weighted EXAFS spectra in the k-space ranging from 2-10.5 $Å^{-1}$ were Fourier-transformed to real (R) space using a hanning window.

Computation methods: l the spin-polarized calculations were performed using the Vienna ab initio Simulation Package (VASP)[1, 2] with projector-augmented waves (PAW) pseudopotentials and the exchange-correlation functionals parametrized by Perdew, Burke, and Ernzerhof for the generalized gradient approximation (GGA)[3-5]. Dispersion-corrected density functional theory calculations (DFT-D3)[6] were performed for group adsorbed on $Co\text{-}N_4/G$ and Co cluster) surfaces. We set an energy cutoff, a convergence criterion for self-consistent iteration and ionic relaxation to be 480 eV, 10^{-5} eV and 0.05 eV $Å^{-1}$, respectively. The k-space integration was performed using a 3 × 3 × 1 Monkhorst-Pack grid. For the catalytic reaction, the model of Co-NC is built by coordinating Co atoms with four nitrogen atoms to form a square planar $Co\text{-}N_4$ structure on nitrogen-doped graphene, as determined from the fitting results of EXAFS data. The cluster model with ten Co atoms placed on nitrogen-doped graphene was applied to represent the $Co_{NP}NC$. The entropy contributions of small molecules (TΔS), including the vibrational, rotational and translational entropies, were considered to estimate the Gibbs free energy change (ΔG) at the temperature of 298 K. The ΔG values of elementary steps were estimated according to

$\Delta G = \Delta H - T\Delta S$. The reaction enthalpy (ΔH) was approximated with the total energy difference (ΔE), neglecting the small zero-point energy correction (ΔZPE), heat capacity correction and $\Delta(pV)$ term.[7]

Supplementary Figures

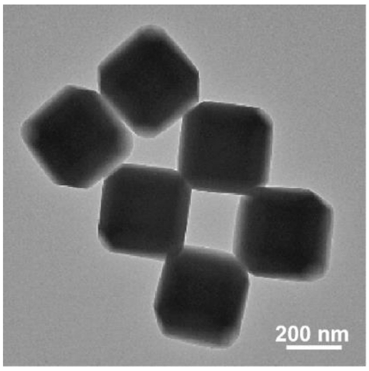

Supplementary Fig. 1 TEM image of ZnCo-BZIF.

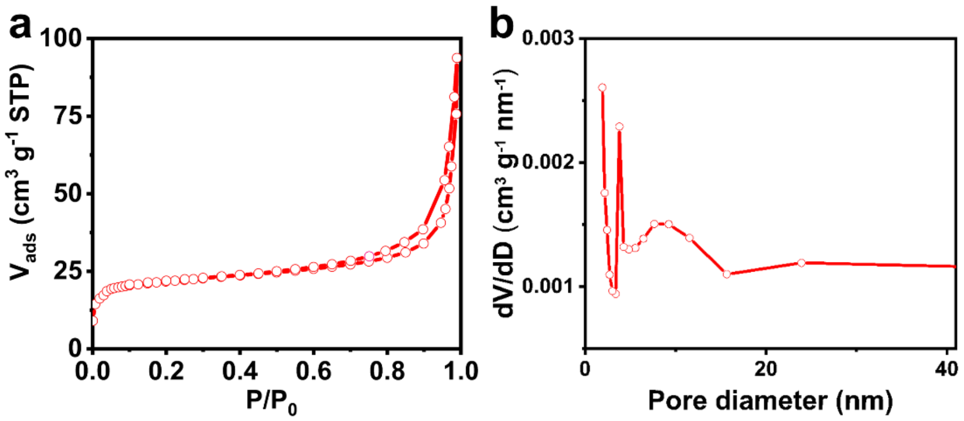

Supplementary Fig. 2 a Nitrogen adsorption and desorption isotherms and **b** pore size distribution of h-BZIF. The relatively small BET value is probably due to the encapsulation of TA on the surface of ZIF.

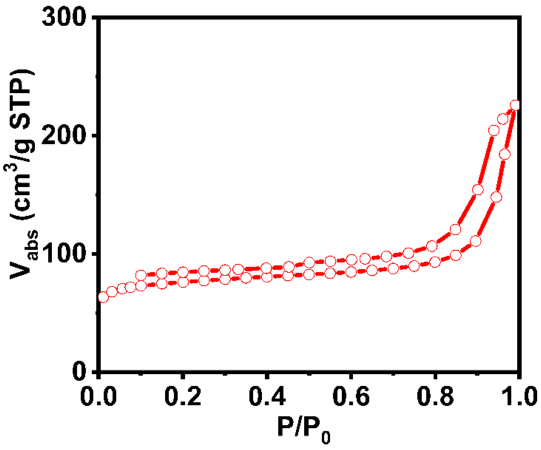

Supplementary Fig. 3 Nitrogen adsorption and desorption isotherm of h-CoNC.

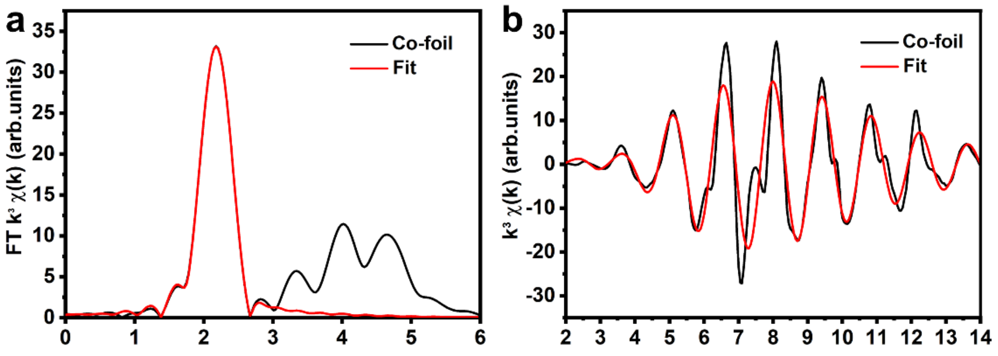

Supplementary Fig. 4 The EXAFS fitting curves of Co foil. a R space and **b** k space.

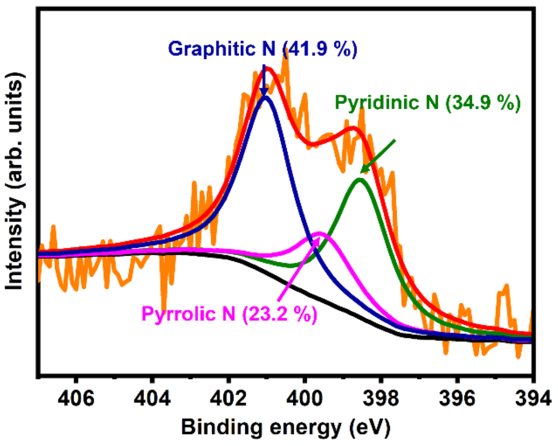

Supplementary Fig. 5 High-resolution XPS spectrum of N 1*s* for h-CoNC. XPS suggested that the nitrogen in h-CoNC was composed of pyridinic nitrogen, pyrrolic nitrogen and graphitic nitrogen, with peaks at 398.5 eV, 399.6 eV and 401.1 eV, respectively.

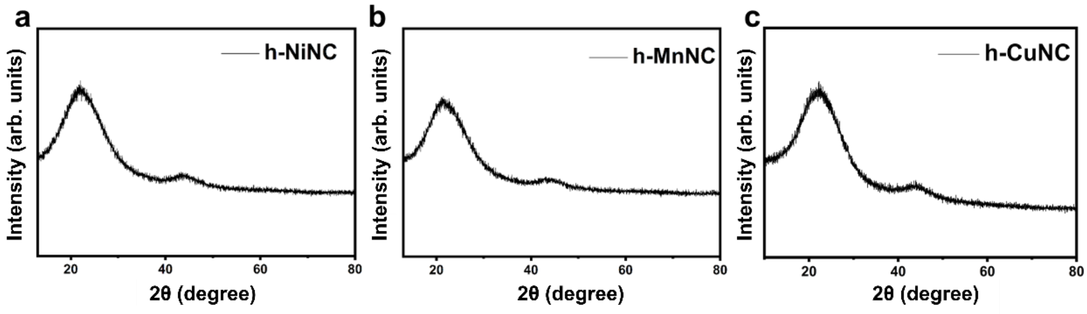

Supplementary Fig. 6 XRD patterns of different h-MNCs. a h-NiNC, **b** h-MnNC and **c** h-CuNC.

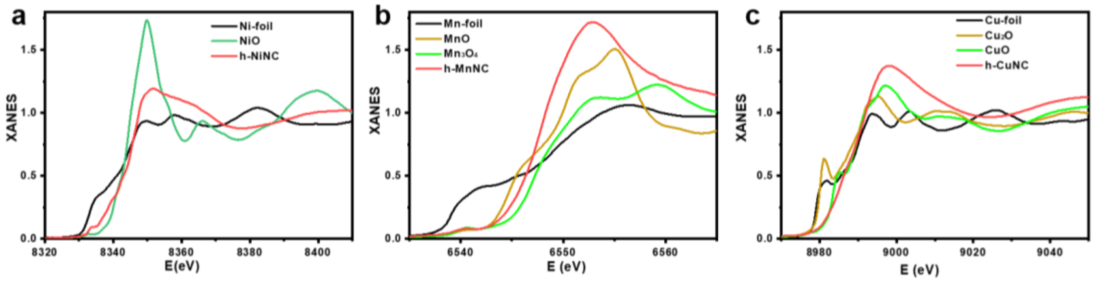

Supplementary Fig. 7 XANES spectra of different h-MNCs. a h-NiNC at the Ni K-edge, **b** h-MnNC at Mn K-edge and **c** h-CuNC at Cu K-edge with their references.

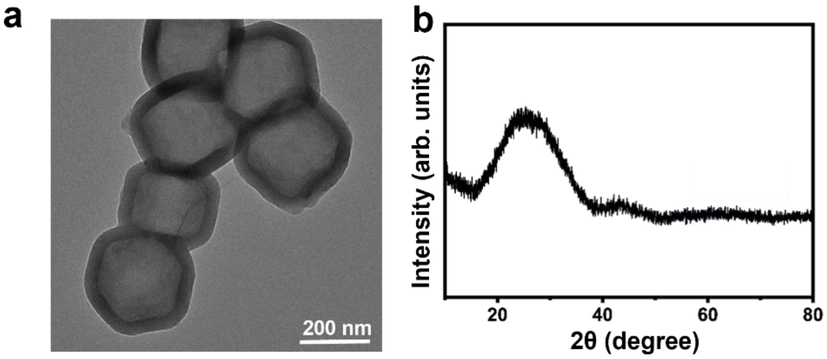

Supplementary Fig. 8 Characterization of h-NC. a TEM image and **b** XRD pattern of h-NC.

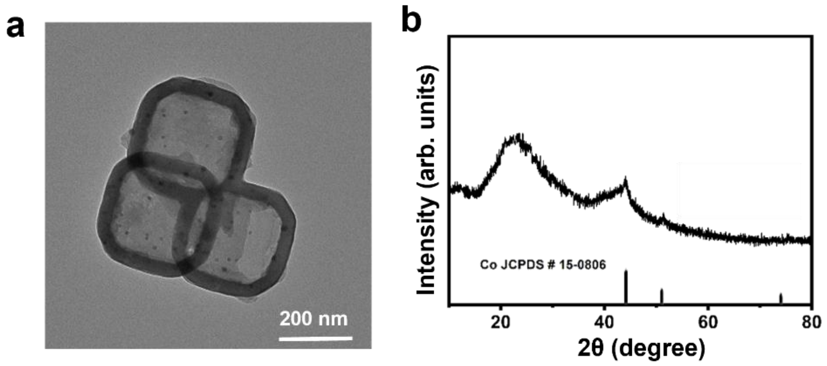

Supplementary Fig. 9 Characterization of h-Co$_{NP}$NC. a TEM image and **b** XRD pattern of h-Co$_{NP}$NC. XRD pattern indicated the presence of metallic Co in h-Co$_{NP}$NC.

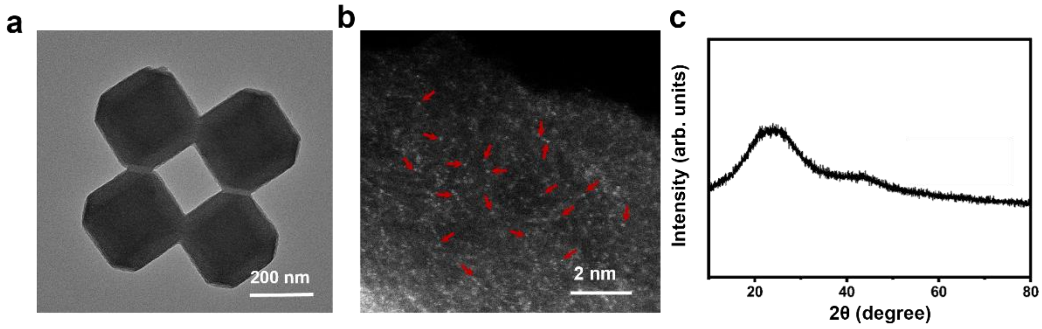

Supplementary Fig. 10 Characterization of s-CoNC. a TEM image, **b** AC-HAADF-STEM image (numerous Co atoms are indicated by red arrows to facilitate identification) and **c** XRD pattern of s-CoNC. Only discrete bright dots were observed on the AC-HAADF-STEM image, demonstrating the formation of atomic dispersion of Co atoms of s-CoNC. XRD pattern of h-CoNC demonstrated the absence of metallic cobalt in s-CoNC.

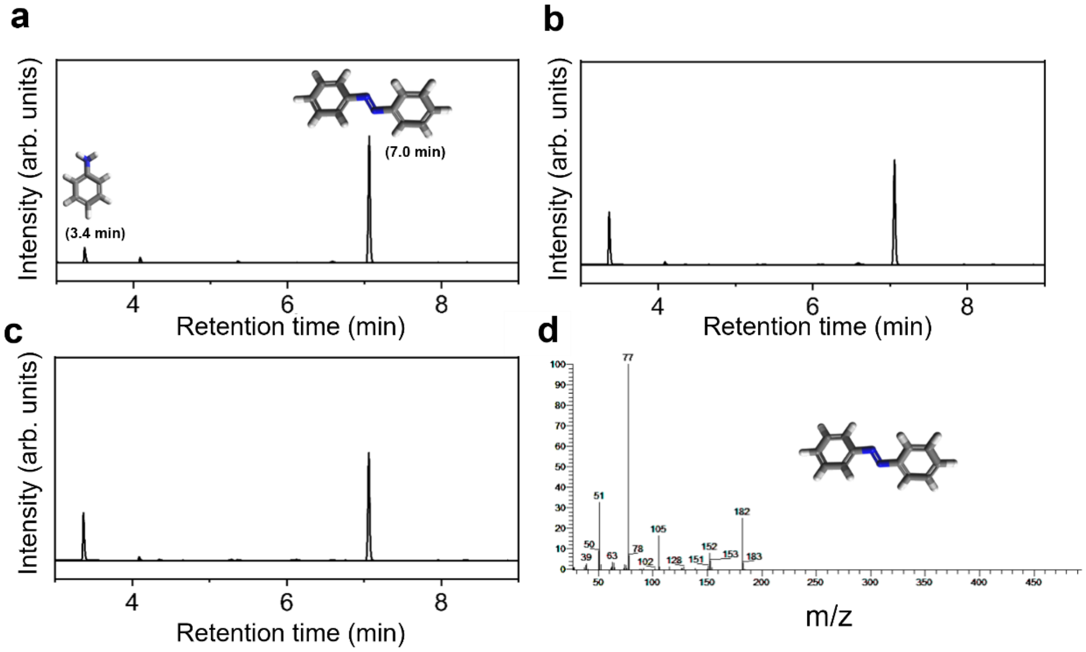

Supplementary Fig. 11 a GC spectra of the products of oxidative coupling of aniline using **a** h-CoNC, **b** s-CoNC and **c** h-Co$_{NP}$NC as catalysts. Only azobenzene (with the retention time of 7.0 min) was observed as the product (The peak at the retention time of 3.4 min was the reactant aniline). **d** Mass spectrum of the product azobenzene.

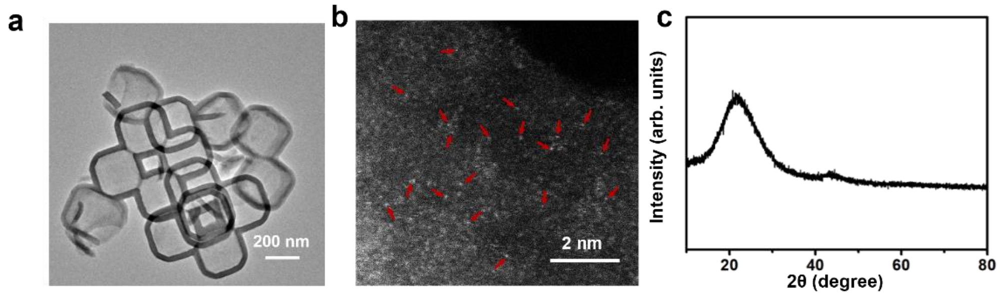

Supplementary Fig. 12 Characterization of reused h-CoNC. a TEM image, **b** AC-HAADF-STEM image (numerous Co atoms are indicated by red arrows to facilitate identification) and **c** XRD pattern of the h-CoNC after five cycles of coupling reactions. No obvious change was observed from the TEM image and XRD pattern of the reused catalysts and the AC-HAADF-STEM image shows atomic disperse of Co atoms, indicating the excellent stability of h-CoNC.

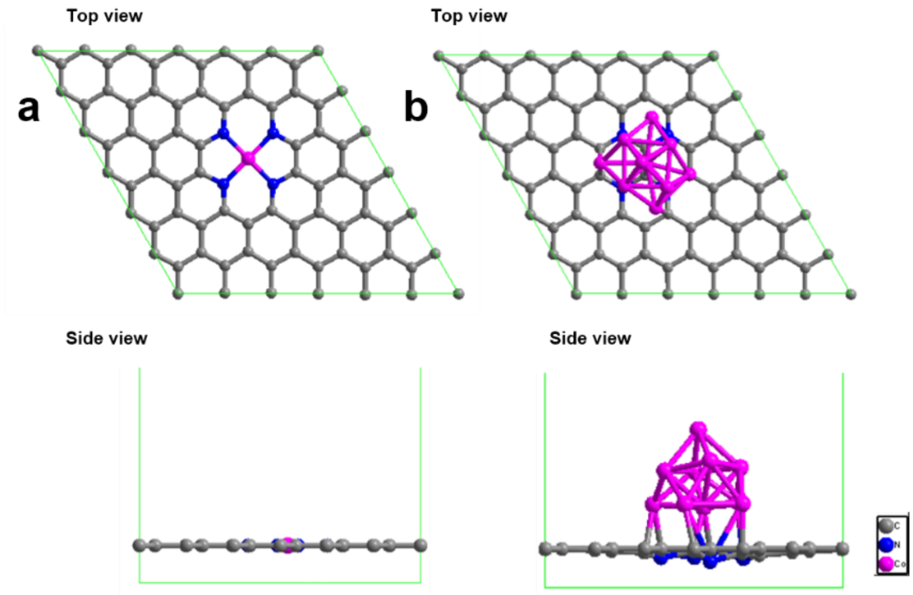

Supplementary Fig. 13 Calculation models of Co-NC and Co$_{NP}$NC. **a** The model of Co-NC constructed by one isolated Co atom bonded with 4 N atoms on graphene planes which substituted 6 carbon atoms. **b** The model of Co$_{NP}$NC constructed by 10 Co atoms bonded to graphene planes as Co particle. A graphene supercell (6 × 6) consisting of 66 carbon atoms with 15 Å of a vacuum layer perpendicular to the graphene sheets was employed as the model of carbon materials.

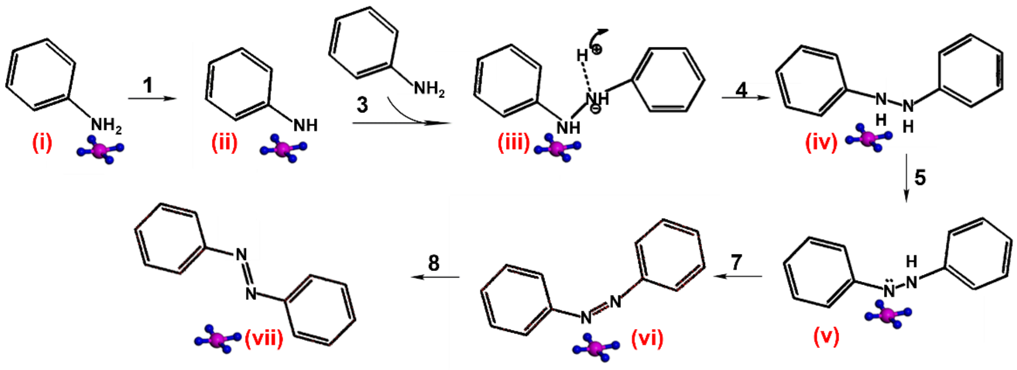

Supplementary Fig. 14 The reaction pathway of aniline coupling to azobenzene.

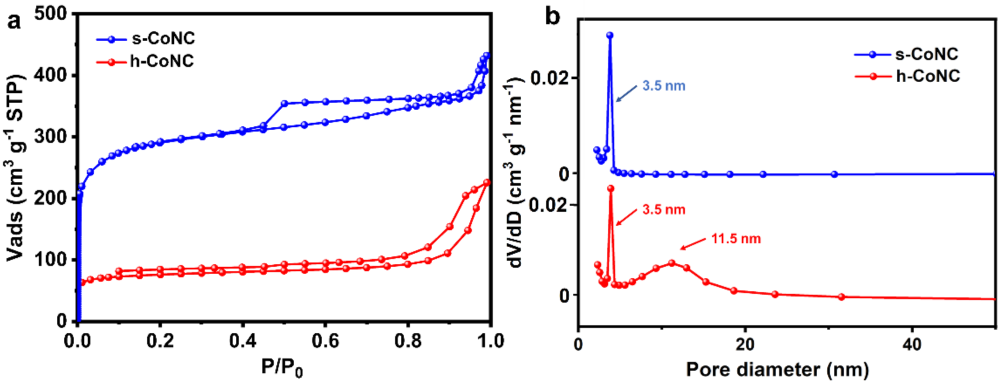

Supplementary Fig. 15 a Nitrogen adsorption and desorption isotherms of h-CoNC and s-CoNC and **b** BJH desorption pore size distribution curves of h-CoNC and s-CoNC.

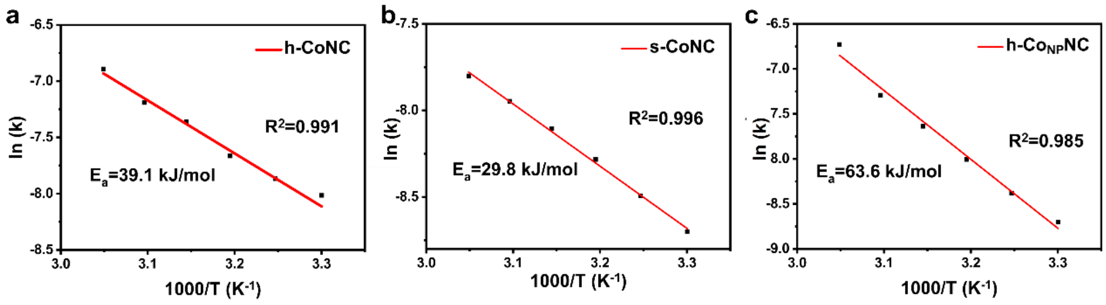

Supplementary Fig. 16 The Arrhenius plots for the aniline coupling over different catalysts. a h-CoNC, **b** s-CoNC, **c** h-Co$_{NP}$NC. Considered the same atomically dispersed catalytic sites of h-CoNC and s-CoNC, the lower E_a means the reaction suffers more internal diffusion barriers. Compared with the same structure of h-CoNC and h-Co$_{NP}$NC, lower E_a of h-CoNC means atomically dispersed catalytic site has a lower reaction barrier than particles site.

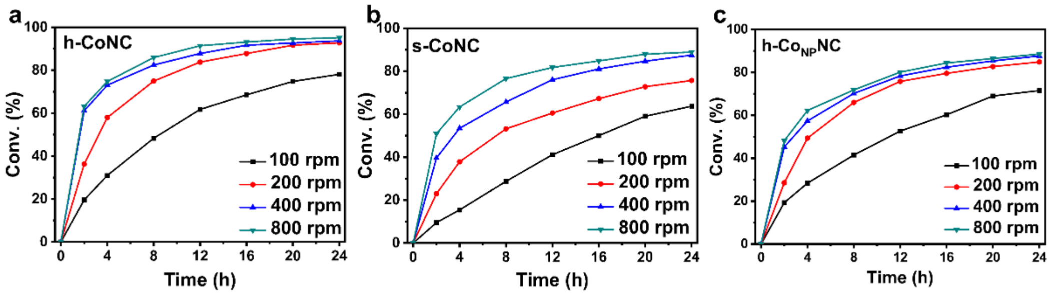

Supplementary Fig. 17 The conversion of different catalysts of coupling of aniline at different stirring rates. a h-CoNC, **b** s-CoNC, **c** h-Co$_{NP}$NC. The conversions increase with the increasing stirring rates because external diffusion was gradually excluded. When the stirring rates were larger than 400 rpm, the effect of external diffusion can be regarded as completely excluded.

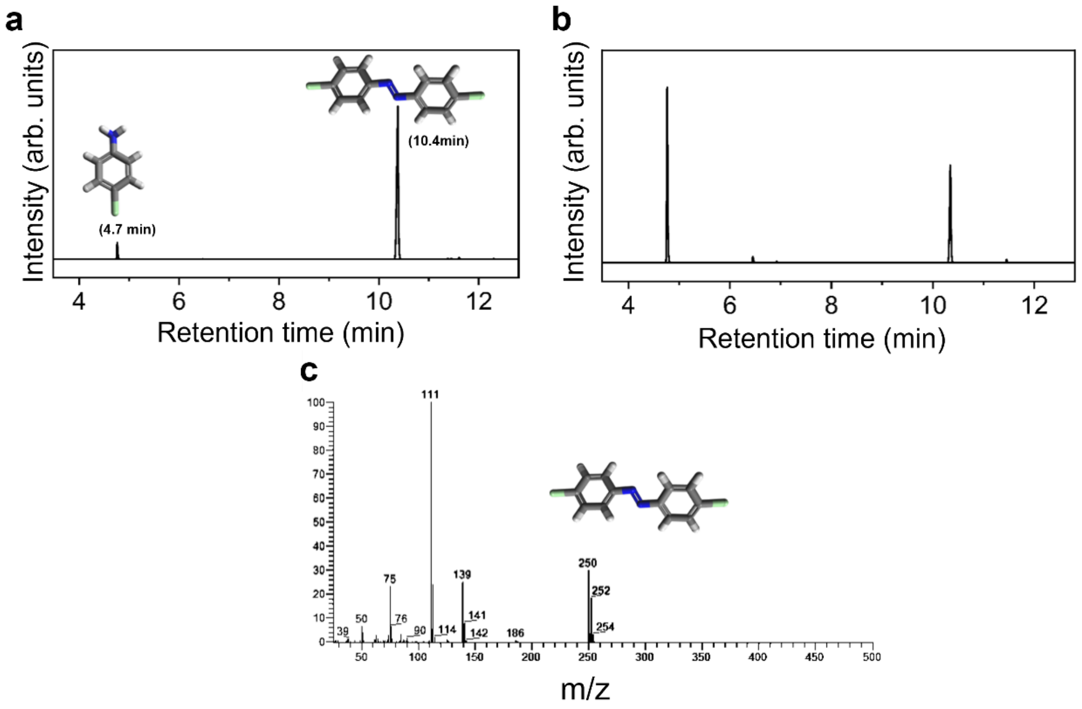

Supplementary Fig. 18 GC spectra of the products of oxidative coupling of 4-chloroaniline using **a** h-CoNC and **b** s-CoNC as catalysts. Only 1,2-bis(4-chlorophenyl)diazene (with the retention time of 10.4 min) was observed as the product (The peak at the retention time of 4.7 min was the reactant 4-chloroaniline). **c** Mass spectrum of the product 1,2-bis(4-chlorophenyl)diazene.

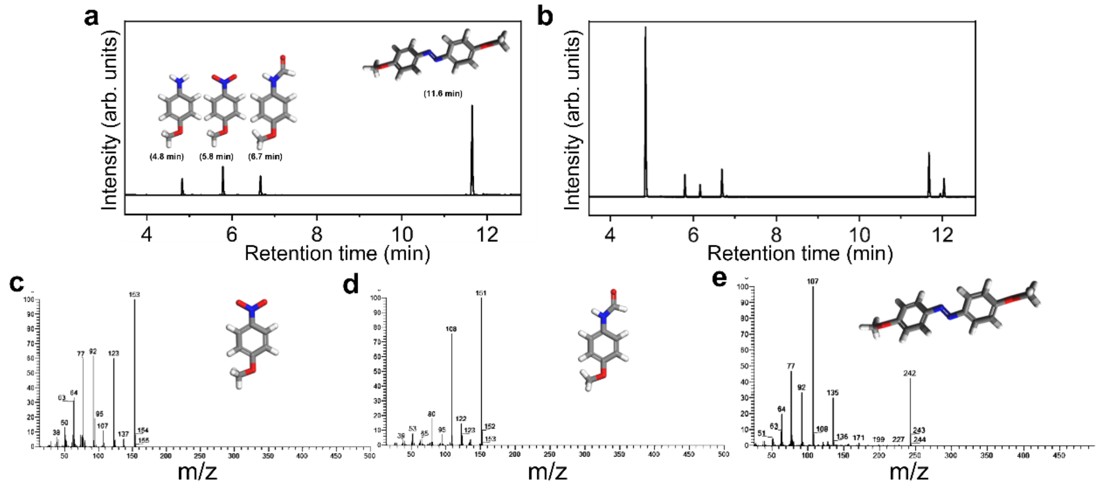

Supplementary Fig. 19 GC spectra of the products of oxidative coupling of 4-methoxyaniline using **a** h-CoNC and **b** s-CoNC as catalysts, and mass spectra of **c** 4-nitroanisole (retention time of 5.8 min), **d** N-(4-methoxyphenyl)formamide (retention time of 6.7 min) and **e** 4,4'-dimethoxyazobenzene (retention time of 11.6 min). The peak at the retention time of 4.8 min was corresponding to the reactant 4-methoxyaniline.

Supplementary Tables

Supplementary Table 1. The content of metal in various h-MNCs (measured by ICP-OES).

Sample	h-CoNC	h-NiNC	h-MnNC	h-CuNC
Metal content (wt.%)	0.42	0.43	0.34	0.10

Supplementary Table 2. BET surface area and the pore volume of different catalysts.

Sample	h-CoNC	s-CoNC	h-Co$_{NP}$NC
BET surface area (m^2 g^{-1})	293.5	938.8	286.9
Volume of micropores (cm^3 g^{-1})	0.11	0.29	0.10
Volume of mesopores (cm^3 g^{-1})	0.27	0.22	0.25

Supplementary Table 3. EXAFS fitting results. Structural parameters of h-CoNC extracted from the EXAFS fitting (S_0^2=0.76). EXAFS results fully demonstrate Co in h-CoNC were coordinated with four nitrogen atoms.

Sample	Scattering Path	CN	R (Å)	σ^2(10^{-3}Å^2)	ΔE_0 (eV)	R factor
Co foil	Co-Co	12*	2.50±0.01	6.4±0.1	8.0±0.2	0.001
h-CoNC	Co-N	3.7±1.2	1.93±0.02	16.7±4.2	-5.3±3.2	0.015

S_0^2 is the amplitude reduction factor; CN is the coordination number; R is the interatomic distance (the bond length between central atoms and surrounding coordination atoms); σ^2 is Debye-Waller factor (a measure of thermal and static disorder in absorber-scatter distances); ΔE_0 is edge-energy shift (the difference between the zero kinetic energy value of the sample and that of the theoretical model). R factor is used to value the goodness of the fitting.

* This value was fixed during EXAFS fitting, based on the known structure of Co foil.

Supplementary Table 4. Comparison of the catalytic activity of aniline oxidative coupling of h-CoNC with other reported catalysts.

Catalyst	Temp. (^oC)	Oxidant	Conv./Select. (%)	Metal content (%)	TOF (h^{-1})	Reference
h-CoNC	RT[a]	O_2	94.4/100	0.42	586	this work
Au/TiO_2	100	O_2 (5 bar)	100/90	1.5	39.2*	Science 2008, 322, 1661.
Ag NPs	25	air	51/100	100	6.7*	ACS Catal. 2013, 3, 478.
Ir(dF-CF_3-ppy)$_2$ (dtbpy)+	24	air	55/100	34.3	2.8*	J. Am. Chem. Soc., 2021, 143, 2938-2943.
RuO_2/Cu_2O NPs	RT	air	94/-	1.78	6.7*	ACS Sustainable Chem. Eng. 2018, 6, 11345-11352.
CuBr	60	O_2	96/-	44.3	5.1*	Angew. Chem. Int. Ed. 2010, 49, 6174.
Meso Mn_2O_3	110	air	99/93	67.0	0.39*	Angew. Chem. Int. Ed. 2016, 128, 2211-2215.
MnOOH	25	O_2	88/100	62.5	1.1*	J. Mater. Chem. A., 2021, 9, 19692-19697.
$BiVO_4$/g-C_3N_4	27	O_2	20/82.3	31.7	0.14*	ACS Sustainable Chem. Eng., 2017, 5,2562-2577.
$[N(C_4H_9)_4]_2[Mo_6O_{19}]$	60	H_2O_2	99/-	42.2	0.01*	Angew. Chem. Int. Ed. 2021, 60, 6382-6385.
$Zr(OH)_4$	100	O_2	95/94	57.3	2.4*	Angew. Chem. Int. Ed. 2022, 134, e202112907.

[a]RT= room temperature

* The conversion and metal content used to calculate TOF were obtained from references.

Supplementary Table 5. Conversion of coupling of 4-chloroaniline.

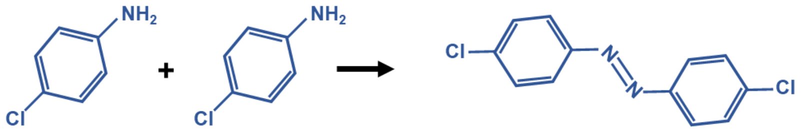

a **a** **(1)**

Catalyst	Conversion	TOF (h^{-1})	Selectivity to (1)	Co (wt.%)
h-CoNC	92.5%	585	96.8%	0.42
s-CoNC	22.5%	142	94.8%	0.47

Supplementary Table 6 Conversion of coupling of 4-methoxyaniline.

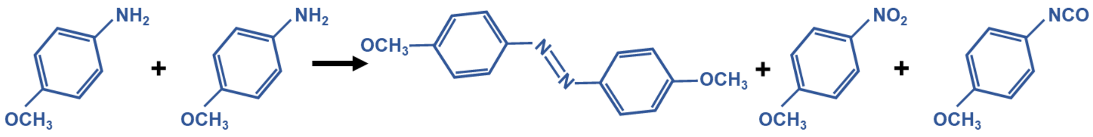

a **a** **(1)** **(2)** **(3)**

Catalyst	Conversion	TOF (h^{-1})	Selectivity to (1)	Selectivity to (2)	Selectivity to (3)
h-CoNC	91.5%	578	80.6%	11.6%	7.8%
s-CoNC	11.3%	58	46.6%	23.2%	22.4%

Supplementary References

1. Kresse G & Furthmüller J. Efficiency of ab-initio total energy calculations for metals and semiconductors using a plane-wave basis set. *Comput. Mater. Sci*. **6,** 15-50 (1996).
2. Kresse G & Joubert D. From ultrasoft pseudopotentials to the projector augmented-wave method. *Phys. Rev. B* **59**, 1758-177 (1999).
3. Kresse, G. & Furthmuller, J. Efficient iterative schemes for ab initio total-energy calculations using a plane-wave basis set. *Phys. Rev. B* **54**, 11169-11186 (1996).
4. Kresse, G. & Hafner, J. Ab initio molecular-dynamics simulation of the liquid-metal-amorphous-semiconductor transition in germanium. *Phys. Rev. B* **9**, 14251-14269 (1994).
5. Kresse, G. & Joubert, D. From ultrasoft pseudopotentials to the projector augmented-wave method. *Phys. Rev. B* **59**, 1758-1775 (1999).
6. Grimme, G., Antony, J., Ehrlich, S.& Krieg, H. A consistent and accurate ab initio parametrization of density functional dispersion correction (DFT-D) for the 94 elements H-Pu. *J. Chem. Phys.* **132**, 154104 (2010).
7. Jin J. et al. Insight into room-temperature catalytic oxidation of nitric oxide by Cr_2O_3: A DFT study. *ACS Catal.* **8**, 5415-5424 (2018).
